# Effect of the COVID-19 Pandemic on Children With SMA Receiving Nusinersen: What Is Missed and What Is Gained?

**DOI:** 10.3389/fneur.2021.704928

**Published:** 2021-09-21

**Authors:** Caterina Agosto, Eleonora Salamon, Luca Giacomelli, Simonetta Papa, Francesca Benedetti, Franca Benini

**Affiliations:** ^1^Pediatric Pain and Palliative Care Service, Department of Women's and Children's Health, Padua University Hospital, Padua, Italy; ^2^Polistudium SRL, Milan, Italy; ^3^Pediatric Training Program, University of Padua, Padua, Italy

**Keywords:** SMA, nusinersen, COVID-19 pandemic, nusinersen infusion delay, PPC

## Abstract

Nusinersen is the first oligonucleotide-based drug that is approved for the treatment of spinal muscular atrophy. In January 2020, the WHO declared COVID-19 a pandemic and nusinersen-provider centers had to postpone planned infusions for some children along with other related interventions. Considering the important contribution that the intrathecal infusions and other support activities could have on the quality of life of spinal muscular atrophy patients and their families, this emergency could have a relevant impact on the course of the pathology. The present work aims to assess the clinical and social issues that arise for spinal muscular atrophy children in care at the referral pediatric palliative care Centre of Padua (Veneto) from a delay in nusinersen infusions, resulting from the contingent COVID-19 restrictions. This evaluation has been carried out in both the short and long term after the first lockdown period and can be considered as a “proxy” of a situation of a possible delay in administration or management of infusions, due to other different causes.

## Introduction

Spinal muscular atrophy (SMA) is a relatively rare neuromuscular disorder, which could lead to infant mortality (1). It is caused by the loss or mutation of the “survival of motor neuron” gene, termed SMN1. This induces the degeneration of motor neurons, with progressive muscle weakness and atrophy (2). There are five subtypes of SMA, characterized by different clinical severity (3).

Nusinersen is the first oligonucleotide-based drug that is approved for the treatment of SMA (4); it has been available in the market in Italy since October 2017 (5, 6). This molecule has shown to be effective in patients with SMA1 and SMA2 in pivotal trials (NCT02193074 and NCT02292537) (7, 8). However, nusinersen requires an intrathecal administration, and the benefits potentially associated with this treatment can be disrupted using invasive procedures (9, 10). Indeed, some patients treated with nusinersen, especially those who started treatment at an older age, do not actually improve their clinical status and remain disabled with a continuous requirement of intensive care, thus raising ethical implications related to this treatment (11, 12).

In January 2020, the World Health Organization (WHO) declared COVID-19 a pandemic (13). Due to the COVID-19 emergency, nusinersen-provider centers had to postpone planned infusions for some children (14).

In the Veneto region of Italy, children diagnosed with SMA are cared for from birth in the pediatric palliative care (PPC) program, which provides coordination of follow-up, enrollment therapy, parental qualification and network coordination (15). In the referral PPC Centre of Padua (Veneto), during the first lockdown period, some activities had to be changed or suspended.

Considering the important contribution that the intrathecal infusions and other support activities (nursing interventions, physiotherapy activity, and medical intervention) could have on the quality of life of patients and their families, stopping them could have a relevant impact.

The present work aims to assess the clinical and social issues that arise for PPC children and their families from a delay in nusinersen infusions, resulting from the contingent COVID-19 restrictions. This evaluation has been carried out both in the short and long term after the first lockdown period and can be considered as a “proxy” of a situation of a possible delay in administration or management of infusions due to other different causes.

## Patients and Methods

### Study Design

In the referral PPC Centre of Padua (Veneto), during the first lockdown period and accordingly to a shared decision taken by all the national nusinersen-provider centers, the nusinersen loading doses were configured as a health emergency and therefore respected, maintenance doses instead were moved in compliance with the restrictions, but not exceeding an interval of 6 months (16).

Consequent to the COVID-19 emergency, clinical and social issues for SMA patients were evaluated through a medical record review that has been carried out during these time points: (1) first diagnosis of SMA; (2) last nusinersen administration before the first lockdown period; (3) first nusinersen administration after the first lockdown period (on time or delayed; short-term evaluation); and (4) second nusinersen administration after the first lockdown period (on time; long-term evaluation).

In addition, a cross-sectional survey has been conducted at the moment of the first nusinersen administration after the lockdown period.

Both tools involved children in care at the regional referral center for PPC of Padua in the Veneto region and their families.

### Study Population

Children (0–18 years) with a genetic diagnosis of SMA in maintenance therapy with nusinersen who consent to participate in the study have been considered. Children performing loading doses, children for whom it has been decided to not continue the therapy with nusinersen, and families with a language barrier have been excluded from the study. The Local Ethical Committee has approved this study (protocol number: 0049524), and all patients, or their caregivers, have signed an informed consent to use the data for research purposes.

### Procedures

Support for SMA patients and families is routinely guaranteed by the PPC center with a telephone availability 24/7 and a nursing telephone monitoring intervention every 15–30 days. During the first lockdown period, extraordinary support was provided by converting physiotherapy activity into video call interventions. Home interventions (for medical need or physiotherapy activity) have been provided only in cases of necessity in compliance with the safety standards.

#### Medical Record Review

For each time point, the following data were collected in a clinical practice setting: weight, height, chest circumference, head circumference (only for children <3 years age), thoracic circumference (17), motor function scale values (CHOP: Children's Hospital of Philadelphia Infant Test of Neuromuscular Disorders, 0–64-point scale composed of 16 items used to assess motor skills (18); HINE: Hammersmith Infant Neurological Examination, a 0- to 26-point scale to evaluate motor skills in children from 2 months to 2 years of age (19); HFMSE: Hammersmith Functional Motor Scale—Expanded, a 0–66-point scale to evaluate motor function in children (20)), and responder or non-responder condition according to the pivotal trials (21–23).

In addition, the number of children for whom the infusion was postponed and how long it was postponed (days) and the number of patients who discontinue infusions have been recorded.

The interventions performed at home or remotely during lockdown by a nurse, physiotherapist, and doctor were also documented for each child.

#### Cross-Sectional Survey

An *ad hoc* questionnaire was administered by two operators of the Center to children (only if ≥6 years) and parents, at the first administration of therapy after the temporary suspension.

The survey contains questions about the perception of muscle strength, swallowing, and breathing functions, along with some questions about personal feelings and fears during the lockdown period.

Improvements relative to muscle strength, swallowing, and breathing have been assessed through a Likert scale, from “not perceived” to “great.”

### Data Analysis

Descriptive statistics were used for the present study, which was performed with an explorative intent.

## Results

### Participants

At the date of discontinuation of intrathecal infusions at the Padua PPC center (27/02/2020), 31 children were on nusinersen therapy. Five out of 31 (16%) patients decided during the lockdown to not resume the therapy and were therefore excluded from the study. One patient has been excluded because of a language barrier. Consequently, a total of 25 children were included in the study. The baseline and clinical characteristics of these children are summarized in Table 1.

**Table 1:** Baseline characteristics of patients.

**Characteristic**	***n* (%)**
Number of considered patients	25 (100)
Females	13 (52)
Age (years), median (range)	8 (2–15)
**Diagnosis**	
SMA1a	1 (4)
SMA1b	1 (4)
SMA1c	7 (28)
SMA2	9 (36)
SMA3	7 (28)
**Functional status**	
Sitter	16 (64)
Layer	8 (32)
Walker	1 (4)
**Ventilation**	
Autonomous	9 (36)
Non-invasive ventilation	15 (60)
Invasive mandatory ventilation	1 (4)
**Family**	
Biparental	23 (92)
Monoparental	2 (8)
**Principal caregiver**	
Mother	24 (96)
Father	1 (4)

### Medical Record Review

#### Overview of the Population

The data for every single patient are reported in Table 2. In total, 11 patients (44%) were responders to nusinersen at the last infusion before lockdown. Of them, one patient had a diagnosis of SMA1a, one of SMA1b, two of SMA1c, five of SMA2, and two of SMA3. Consequently, responder rates were 100% (1/1) for SMA1a, 100% (1/1) for SMA1b, 28% (2/7) for SMA1c (24), 55% (5/9) for SMA2, and 28% (2/7) for SMA3. Among all responders, eight (73%) received early treatment (i.e., within 1 week since the genetic diagnosis of SMA); seven (64%) were sitters, and four (36%) were layers; three (27%) were on autonomous ventilation, seven (64%) were on NIV (non-invasive ventilation), and one (9%) required invasive mechanical ventilation. Four of the responders (36%) had poor compliance to the standard of care, defined according to Finkel et al. (25).

**Table 2:** Patient data overview.

**Case label**	**Age (years)**	**SMA type**	**Functional level**	**Early treatment**	**Scale for assessment**	**Score at diagnosis**	Score at last infusion^*^	Responder^*^	**Score at 1st infusion^**§**^**	**Delta 1st infusion vs. pre-lockdown**	**Score at 2nd infusion^**§**^**	**Delta 2nd vs. 1st infusion^§^**	**Delta 2nd infusion vs. pre-lockdown**	**Days of delay**
021	4	1a	Layer	Yes	HFMSE	23	40	Yes	37	−3	38	1	−2	0
009	3	1b	Layer	Yes	CHOP	23	40	Yes	39	−1	41	2	1	57
004	3	1c	Layer	Yes	CHOP	29	47	Yes	45	−2	NA	-	-	0
005	3	1c	Sitter	Yes	CHOP	50	50	No	52	2	52	0	2	26
006	5	1c	Layer	Yes	CHOP	44	41	No	36	−5	40	4	−1	0
008	5	1c	Layer	Yes	CHOP	35	34	No	34	0	30	−4	−4	91
023	12	1c	Layer	No	CHOP	13	11	No	11	0	-	-	-	-
024	10	1c	Sitter	No	CHOP	43	33	No	33	0	32	−1	−1	91
030	11	1c	Layer	No	HFMSE	20	27	Yes	27	0	23	−4	−4	0
001	3	2	Sitter	Yes	HFMSE	10 (HINE)	28	Yes	30	2	33	3	5	44
002	3	2	Sittaaer	Yes	HINE	8	17	Yes	15	−2	15	0	−2	30
003	3	2	Sitter	Yes	HFMSE	9 (HINE)	27	Yes	31	4	32	1	5	0
011	9	2	Layer	No	HFMSE	4	6	No	5	−1	5	0	−1	0
012	4	2	Sitter	Yes	HFMSE	24	43	Yes	44	1	NA	-	-	0
014	13	2	Sitter	No	HFMSE	9	13	Yes	13	0	13	0	0	0
015	8	2	Sitter	No	HFMSE	10	8	No	9	1	10	1	2	0
018	12	2	Sitter	No	HFMSE	17	7	No	9	2	8	−1	1	0
031	10	2	Sitter	No	HFMSE	5	5	No	5	0	5	0	0	0
007	6	3	Sitter	Yes	HFMSE	41	45	Yes	44	−1	44	0	−1	0
010	9	3	Walker	Yes	HFMSE	61	63	No	62	−1	63	1	0	73
013	14	3	Sitter	No	HFMSE	32	31	No	24	−7	24	0	−7	59
019	11	3	Sitter	No	HMFSE	46	42	No	42	0	43	1	1	0
022	12	3	Sitter	No	HFMSE	36	30	No	30	0	30	0	0	0
025	15	3	Sitter	No	HFMSE	10	8	No	8	0	6	−2	−2	0
026	7	3	Sitter	No	HFMSE	32	35	Yes	37	2	42	5	7	0

* *Before lockdown period.*

§ *After lockdown period*.

#### First Visit After Lockdown

Considering all 25 patients, 9 patients (36%) showed a reduction in the functional score at the first visit after the lockdown period, compared with the last visit before the lockdown period, whereas five patients (20%) showed an improvement. Among the 9 patients who complained of a reduction in the functional score, no one was a responder. Only in three cases was the reduction in the functional score >-2: one patient with poor compliance to the standard of care (patient number 6, −5 points on the CHOP scale), one patient aged 4 years with a natural history of more severe disease (patient number 21, −3 points on the HFMSE scale), and one patient aged 14 years with a marked worsening of scoliosis (patient number 13, −7 points on the HFMSE scale).

A total of eight children (32%) experienced a delay in the infusion schedule (median delay, 58 days; range, 26–91). Among them, only marginal changes in the functional scores were reported, except for patient number 13 (see above) who had a 59-day delay in the administration of nusinersen.

During the lockdown, each family received two phone calls a month from the nurses. A total of 100 nursing calls have been carried out.

The physiotherapist made a total of 64 video calls to patients with a median of two calls per patient (range: 1–5) and six home visits.

During the lockdown, no children needed to access the emergency room or were hospitalized.

#### Second Visit After Lockdown

Long-term evaluations were collected at the second infusion after lockdown, which has been administered 4 months after the previous infusion without delay and has been compared with the last visit before the lockdown period. A total of 10 patients (40%) showed a reduction in the functional score, whereas 8 patients (32%) showed an improvement. Only in three cases was the reduction in the functional score >-2: patient 13, who did not improve his score compared with the previous evaluation (−7 points on the HFMSE scale, see above), one patient with a single and fatigued parent as a sole caregiver (patient 8, −4 points on the CHOP scale), and one patient with a difficult social situation and parents who experienced depression due to the loss of their jobs (patient 30, −4 points on the CHOP scale). Both patients 8 and 30 got worse between the first and second infusions after lockdown.

Patient 26 experienced a great improvement in his score, with a 7-point increase since the pre-lockdown evaluations. This was due to the presence of two caregivers during the lockdown.

Of note, patient 6, who experienced a great score loss at the first visit after lockdown (see above), improved his score by 4 points at this second evaluation: this was due to better compliance with supportive therapies (cough machine).

### Questionnaires

#### Parents

In total, 25 parents were interviewed; the responder was primarily the mother (23 out of 25, 92%).

**Figure 1:**
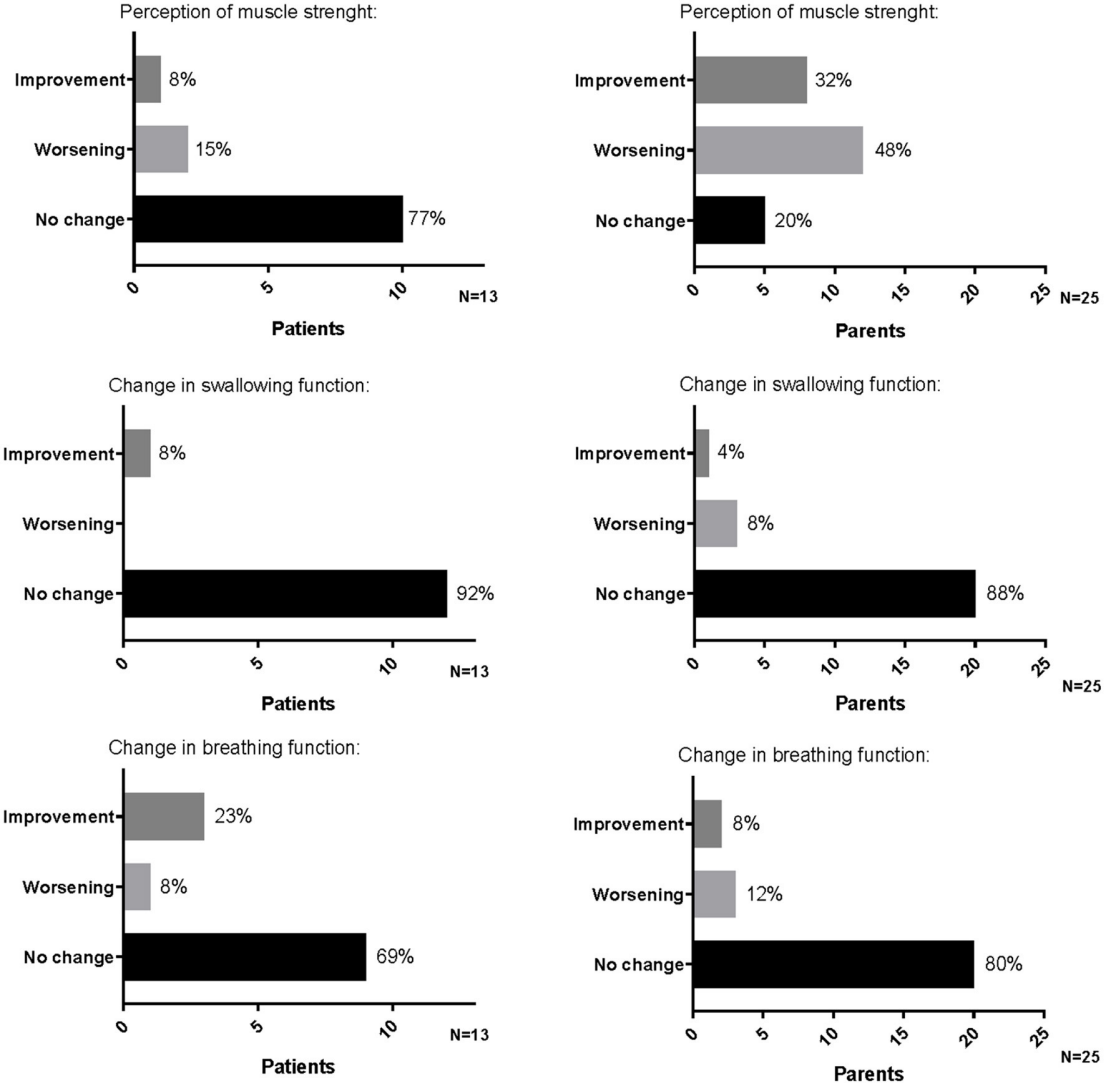
Perception of muscle strength, swallowing, and breathing functions reported by patients and parents on the occasion of the first administration of therapy after the temporary suspension.

Among the parents interviewed, 5 out of 25 (20%) did not perceive a change in their child's muscle strength, 12 out of 25 (48%) felt a worsening (one rated the worsening as great, five as quiet, and the other as small/very small), and 8 out of 25 (32%) felt an improvement (two rated the improvement as great, five as quiet, and one as very small) (Figure 1).

A total of 22 parents (88%) did not perceive a change in the child swallowing function, 2 parents (8%) felt a worsening (one rated as great and one as very small), and 1 (4%) felt a great improvement (Figure 1).

A total of 20 parents (80%) did not perceive a change in child breathing function, 2 parents (8%) felt a great improvement, and 3 (12%) felt a worsening rating as “quiet” and had to intensify the respiratory support to their children (Figure 1).

Parents retain that worsening changes are mainly due to the suspension of outpatient physiotherapy (10 out of 25, 40%) or to a delay in nusinersen infusion (6 out of 25, 24%). Six out of 25 (24%) are unable to identify a cause, two (8%) parents attribute the worsening to the impossibility of changing aids, and one (4%) attributes it to less home help.

Improving changes are attributed to less fatigue for the child in six cases (24%), to the time spent with the family (six cases, 24%), and to the adjunctive home respiratory physiotherapy (six cases, 24%) or stretching (five cases, 20%); two parents (8%) failed to identify a reason.

Three parents (12%) said that they were not worried about the interruption of therapy; the rest said they were quite worried (three cases, 12%), slightly worried (five cases, 20%), very worried (eight cases, 32%), and extremely worried (six cases, 24%).

The final question concerns the assistance received during the lockdown period: 12 parents (48%) report to have not requested any additional help compared to what is normally provided and 13 parents (52%) report to have benefited from the extraordinary support of the PPC center.

#### Children

Among the 25 children of the study, 14 were >6 years. One did not consent to participate; consequently, a total of 13 children were interviewed. The median age was 10 years (range: 7–15); 1 was functionally a layer, 11 were sitters, and 1 was a walker.

Among them, 10 (77%) did not perceive a change in their muscle strength, 2 (15%) felt a worsening (reported as small worsening), and 1 (8%) felt a great improvement (Figure 1).

In total, 12 children (92%) did not perceive a change in swallowing function; the other one (8%) felt an improvement, reported as great (Figure 1).

Nine children (69%) did not perceive a change in breathing function, three (23%) felt an improvement (two reported as quite an improvement and one as a great improvement), and one (8%) felt a small worsening with a consequent increase of the assisted ventilation (Figure 1).

Seven children out of 13 (53%) said they were not worried about the interruption of intrathecal therapy; the other said they were quite worried (3/13, 23%), slightly worried (1/13, 8%), very worried (1/13, 8%), and extremely worried (1/13, 8%).

What children missed more during the lockdown period was going to school and seeing their friends in eight cases (61%) or taking lessons in three cases (23%). Two children (16%) missed going to physiotherapy or hydrotherapy more.

## Discussion

The present work aimed to investigate the effects of the delay of nusinersen infusions due to the COVID-19 pandemic on children with SMA, in the context of global care at the PPC center.

Although a detailed statistical analysis was not possible due to the small sample size, no correlation between a delayed treatment and changes of functional scores emerged over the short-period evaluations (~2 months after the end of the first lockdown) and the immediately following months (long-term evaluations).

Furthermore, three patients showed a relevant (>-2) change in functional scores in the short period. This worsening had a strong clinical reason; one patient was not compliant with the standard of care due to a poor social context, the second was a 14-year-old patient with scoliosis, which severely worsened during the last months before the lockdown period, and the third presents a more severe disease trajectory.

When assessing the proportion of responders before the lockdown period, it emerged that the majority of them (approximately three out of four) had an early initiation of treatment, immediately after the genetic confirmation of diagnosis. For the others, the diagnosis preceded the approval of nusinersen therapy for SMA patients. However, no correlation with the changes in functional scores was possible since all score variations reported over the period of the first lockdown were of modest magnitude.

Considering long-term evaluations, 10 patients reported an overall worsening of their functional score compared to evaluations before the lockdown. The reduction was >-2 in three cases. One was the same patient who reported a worsening already in the short term; the other two patients got worse between the first and second infusions after lockdown.

Consequently, the great worsening was seen in both the short- and long-term evaluations and cannot be attributed to the delay in infusions but to a difficult family situation of the patients.

Of note, two patients experienced a global great improvement in the long-term evaluation (+5 points) compared to evaluations before the lockdown, and this was due to great family support.

Considering the survey results, the global patient's perception is to not have suffered a worsening in muscle strength, swallowing, and breathing functions. Otherwise, a worsening in muscle strength is perceived by 48% of parents. This discordant perception is mainly due to a state of anxiety of parents related to the suspension of physiotherapy (reported by 40% of parents).

On the other hand, a worsening of breathing functions is perceived in a minority of cases.

This is because, in our reality, parents are enabled to the home management of the respiratory aspect, supported with 24/7 nurse telephone availability and the possibility of video calls supporting the respiratory physiotherapy.

Of note, considering that a direct consequence of the worsening of muscle strength is an impairment of the breathing function, this underlines the importance of home physiotherapy.

This is confirmed by the fact that children who had both parents caring for them and helping each other during the lockdown showed the best improvement in motor function, while in the presence of social issues, above all in single-parent families, children suffered a lack of home institutional help.

On the other hand, patients who were facing worsening scoliosis during the lockdown showed even more detrimental evolution in motor function.

For instance, the study period is too short to perceive a change in swallowing functions, as reported in the literature (21).

The PPC center is equipped to provide remote assistance if necessary, and during the pandemic, this kind of assistance was implemented. For most parents (52%), the extraordinary support implemented during the pandemic has been the main source of help. For the other families, the assistance measures routinely carried out were satisfactory even in this emergency period.

## Data Availability Statement

The raw data supporting the conclusions of this article will be made available by the authors, without undue reservation.

## Ethics Statement

The studies involving human participants were reviewed and approved by the Comitato Etico per la Sperimentazione Clinica e Servizio di Bioetica dell'Azienda Ospedale Università Padova and all patients, or their caregivers, have signed an informed consent to use the data for research purposes. Written informed consent to participate in this study was provided by the participants' legal guardian/next of kin.

## Author Contributions

CA, ES, FBene, and FBeni: study design. CA, LG, and SP: manuscript writing. All authors: manuscript editing, approval to submit, data collection, and interpretation.

## Conflict of Interest

LG and SP were employed by the company Polistudium Srl. The remaining authors declare that the research was conducted in the absence of any commercial or financial relationships that could be construed as a potential conflict of interest.

## Publisher's Note

All claims expressed in this article are solely those of the authors and do not necessarily represent those of their affiliated organizations, or those of the publisher, the editors and the reviewers. Any product that may be evaluated in this article, or claim that may be made by its manufacturer, is not guaranteed or endorsed by the publisher.
